# A Novel Synchronization Scheme Based on a Dynamic Superframe for an Industrial Internet of Things in Underground Mining

**DOI:** 10.3390/s19030504

**Published:** 2019-01-26

**Authors:** Aiping Tan, Yuhuai Peng, Xianli Su, Haibin Tong, Qingxu Deng

**Affiliations:** 1 School of Computer Science and Engineering, Northeastern University, Shenyang 110819, China; aipingtan@126.com (A.T.); xianlis@sina.com (X.S.); tong6445@126.com (H.T.); dengqx@mail.neu.edu.cn (Q.D.); 2 Key Laboratory of Vibration and Control of Aero-Propulsion System of Ministry of Education, Northeastern University, Shenyang 110819, China

**Keywords:** Clock synchronization, Industrial Internet of Things (IIoT), hybrid topology, dynamic superframe, underground mining

## Abstract

The Industrial Internet of Things (IIoT) has a wide range of applications, such as intelligent manufacturing, production process optimization, production equipment monitoring, etc. Due to the complex circumstance in underground mining, the performance of WSNs faces enormous challenges, such as data transmission delay, packet loss rate, and so on. The MAC (Media Access Control) protocol based on TDMA (Time Division Multiple Access) is an effective solution, but it needs to ensure the clock synchronization between the transmission nodes. As the key technology of IIoT, synchronization needs to consider the factors of tunnel structure, energy consumption, etc. Traditional synchronization methods, such as TPSN (Timing-sync Protocol for Sensor Networks), RBS (Reference Broadcast Synchronization), mainly focus on improving synchronization accuracy, ignoring the impact of the actual environment, cannot be directly applied to the IIoT in underground mining. In underground mining, there are two kinds of nodes: base-station node and sensor node, which have different topologies, so they constitute a hybrid topology. In this paper, according to hybrid topology of unground mining, a clock synchronization scheme based on a dynamic superframe is designed. In this scheme, the base-station and sensor have different synchronization methods, improving the TPSN and RBS algorithm, respectively, and adjusts the period of the superframe dynamically by estimating the clock offset. The synchronization scheme presented in this paper can reduce the network communication overhead and energy consumption, ensuring the synchronization accuracy. Based on theCC2530 (Asystem-on-chip solution for IEEE 802.15.4, Zigbee and RF4CE applications), the experiments are compared and analyzed, including synchronization accuracy, energy consumption, and robustness tests. Experimental results show that the synchronization accuracy of the proposed method is at least 11% higher than that of the existing methods, and the energy consumption can be reduced by approximately 13%. At the same time, the proposed method has better robustness.

## 1. Introduction

The Industrial Internet of Things (IIoT) is an important application form of the Internet of Things (IoT), providing various services in different fields, such as manufacturing supply chain management, production process optimization, production equipment monitoring and management, environmental monitoring and energy management, industrial safety management, and so on. In recent years, due to the characteristics of IIoT low energy consumption, low transmission rate, and short distance transmission, many closed construction environments, such as mining, tunnels, subways, indoor buildings, etc., using IIoT to achieve data acquisition and transmission for wireless data transmission has become a very meaningful application scenario [1].

In recent years, there are many research results of clock synchronization schemes for industrial Internet of Things, mainly for robustness, synchronization accuracy, and other indicators of algorithm design, in order to meet the requirements of high reliability and high real-time in industrial environments [2]. A clock synchronization scheme for industrial environment is proposed in [3], and Lora technology is used to realize accurate TDMA synchronization. This method can be used to correct the clock offset effectively and has good robustness. A clock synchronization method based on LoRaWAN (Long Range Wide Area Network) standard is proposed in [4], which solves the synchronization problem between gateway and terminal equipment and improves the scalability of the network. This method is suitable for distributed Internet of Things applications, can reduce the cost, and has good performance in packet loss rate and throughput. In addition, there are methods for specific industrial applications, such as industrial robotics [5] and vehicular systems [6].

Underground mining construction is an industry with safety challenges in China. Coal mines, gold mines, and other mining work need to enter the underground roadway for operation, geological environment, and man-made factors lead to the frequent occurrence of mine accidents. For this reason, safety monitoring of IIoT for the underground construction environment is of great significance. In underground mining, the environmental factors of space, temperature, ventilation, noise, and so on are significant challenges for the reliability and real-time of data transmission. For example, in the construction environment of a metallic mine, the non-standard tunnel distribution, the long working distance, and the insufficient energy supply, these factors make the traditional wireless sensor network technology unable to be applied effectively, such as Wi-Fi [7], Wireless-Hart [8], etc. There are a large number of technologies based on the IEEE 802.15.4standard which are suitable for WSNs in underground mining, such as ZigBee [9]. However, the CSMA/CA mechanism adopted by ZigBee cannot realize reliable data transmission under the condition of large data transmission, so the wireless transmission scheme based on TDMA is adopted more and more. A TDMA algorithm for an underground network topology structure is proposed in [10]. As the key technology to ensure the efficiency of TDMA data transmission, wireless clock synchronization technology for WSNs in underground mining is one of the major studies in the field of IIoT. IIoT in underground mining is composed of sensors and convergence equipment with specific needs. The communication distance of the equipment is limited and energy consumption needs to be considered. Currently, there are few wireless clock synchronization methods for underground mining construction environment. The existing methods mainly focus on how to improve the accuracy of clock synchronization, and do not consider the impact of special topology on the synchronization effect, at the same time, do not consider the energy consumption and other factors.

In this paper, a clock synchronization scheme based on a dynamic superframe for IIoT in an underground mine construction environment is proposed. This scheme designs a synchronization mechanism for different types of nodes. It uses as few synchronization messages as possible to realize the clock synchronization of nodes, ensuring accuracy and reducing energy consumption. The proposed method has the following characteristics:

(1) According to the characteristics of underground tunnel of metallic mine, a hybrid network topology is proposed. All base-station nodes (sink nodes) are constructed into a tree topology, and each base-station is taken as the center, which constitute a single-hop star network structure with several sensor nodes.

(2) Clock synchronization is started by the base-station of the root node, and synchronizes each base-station in turn using a hierarchical traversal. According to hybrid topology, there are two types of clock synchronization: the base-station to base-station and the base-station to sensor, which have design different synchronization mechanisms for each type.

(3) In order to solve the problem of clock chip synchronization precision degradation caused by energy consumption reduction, dynamic superframe is used to synchronize the clock. The superframe is divided into three parts: clock synchronization, data transmission, and clock offset analysis. According to the clock offset analysis results, the superframe size is dynamically adjusted to ensure that the clock synchronization results are not affected by the changes of the environment.

The rest of the paper is organized as follows: Section 2 discusses the related work. Section 3 introduces the definition of the system model and related formulas. Section 4 presents the synchronization scheme for WSNs in underground mining. Section 5 presents the method of the experiment and analysis of the result. Section 6 concludes the paper.

## 2. Related Works

IIoT is the application of traditional wireless sensor network in industrial environment; so many current clock synchronization methods are improved based on wireless sensor network technology, and most of the current wireless sensor network clock synchronization methods are designed based on special environments and applications. A clock synchronization method for distributed wireless sensor networks (DWSNs) is proposed in [11]. This method has good robustness and low energy consumption, and is very suitable for large-scale applications of sensor networks. A synchronization method for monitoring node logs in distributed network is proposed in [12], which achieves global synchronization on distributed monitoring platform. Upadhyay, D. et al. [13] proposes a statistical tool for selecting the reference clock offset of time synchronization protocol based on the maximum probability theory, which is based on the competitive message sensing mechanism and adopts a bi-directional message transmission scheme. Different clock synchronization algorithms have different performance on different platforms [14]. Therefore, many traditional methods cannot be directly applied in special environments, which leads to many clock synchronization technologies still being based on the traditional wireless synchronization technology for application and improvement.

At present, there are three types of main wireless clock synchronization methods: receiver-to-receiver synchronization (RRS); sender-to-receiver synchronization (SRS), and receiver-only synchronization (ROS), in which the representative ones are RBS [15], TPSN [16], and FTSP [17]. In [18], and according to three typical clock synchronization models, six different clock synchronization algorithms are proposed, and their performances are compared and analyzed. According to different application scenarios, a new synchronization algorithm can be designed based on the above six algorithms.

### 2.1. Receiver-to-Receiver Clock Synchronization (RRS)

RRS is mainly the interaction of synchronization messages between different nodes in the receiver. Typically, a synchronous beacon on the sending side is required, and the receiving side then exchanges beacon messages with each other and calculates the clock offset. A method applied to real-time clock synchronization is proposed in [19], which is based on the IEEE 802.11 standard and realizes continuous clock synchronization on the basis of message packet loss tolerance. A synchronization method based on an adaptive clock is proposed in [20]. This method describes the probability calculation method of clock synchronization, which mainly trades the clock synchronization precision and resource requirement, and realizes the maximum utilization of resources under the condition of guaranteeing the clock synchronization constraint. A clock synchronization method for small intelligent sensor networks is proposed in [21]. This method can guarantee the clock synchronization accuracy within a certain error range in a certain period of the whole network.

### 2.2. Sender-to-Receiver Synchronization (SRS)

Since the classical algorithm cannot be applied to sparse Ad Hoc networks, a missing synchronization method for sparse ad hoc networks is proposed in [22], which can satisfy the synchronization accuracy under certain constraints. A clock synchronization method for wireless sensor networks based on delay computation is proposed in [23]. The method can be applied to single-hop or multi-hop networks, and the whole network synchronization can be realized by broadcast mode with less energy consumption. A global synchronization method based on sensor networks is proposed in [24], which includes synchronous and asynchronous systems’ synchronization methods. At the same time, it is proved that this method has very good fault tolerance and can guarantee the global clock synchronization accuracy in the presence of malicious nodes. An energy-sensitive wireless clock synchronization method is proposed in [25], which not only pursues accuracy, but also reduces energy at the expense of accuracy.

### 2.3. Receiver-Only Synchronization (ROS)

The purpose of the ROS method is to reduce the amount of information transmission required for wireless clock synchronization, reduce energy consumption, and ensure clock synchronization accuracy. ROS method is mainly applicable to the whole network synchronization, and some nodes can achieve synchronization by listening to broadcast synchronization messages, without additional communication overhead. The classical algorithm of ROS is FTSP, many research results are based on this method to improve and optimize. A wireless clock synchronization scheme based on energy consumption is proposed in [26]. This scheme can improve and optimize the performance based on FTSP protocol, and reduce the energy consumption on the premise of ensuring the clock synchronization.

At present, the main goal of the research on clock synchronization in wireless sensor networks is to improve the synchronization accuracy. Some methods mainly aim at the characteristic of wireless sensor network, realizing the pairwise synchronization through multiple synchronization data transmitting and receiving, ensuring the accuracy of clock synchronization by estimate the clock offset and the clock skew. [27,28,29] present methods with high synchronization accuracy, but these methods are over-dependent on hardware conditions and inadequate consideration of environmental factors. The research results of [30,31,32] show that the low-energy sensor hardware devices usually use crystal oscillator to achieve timing, but its accuracy being limited. With the change of the environment, it will lead to the increase of clock offset. Djenouri, D.et al. [33] summarizes the related work of the clock synchronization technology of WSNs in recent years, and there is less research work on the underground construction environment at present.

In recent years, the research on clock synchronization of wireless sensor networks for industrial construction site has become a research hotspot. In [34], the advantages and disadvantages of the existing clock synchronization algorithms for wireless sensor networks are analyzed in view of the low energy consumption and low speed of wireless sensor networks. A clock synchronization method for IIoT is proposed in [35] which is mainly applied to long-distance clock synchronization and improving the synchronization accuracy. However, it cannot be applied to short-distance and low-energy WSNs. A method for synchronization based on ambient temperature variation is proposed in [36]. This method establishes the mapping relation between environment and clock offset, which reduces communication overhead to a certain extent. However, the method relies on the analysis of a large number of node data and lacks effective analysis of time variation. SHM (structure health monitoring) is a widely used technology in the field of industrial manufacturing at present. The key technologies of SHM wireless sensor networks are studied in [37], in which the clock synchronization problem is analyzed. Wei-Dong, X.U.et al. [38] presents a coalmine-based clock synchronization algorithm for wireless sensor networks, which is based on linear network topology. It analysis TPSN algorithm and improves the traditional clustering algorithm. However, this algorithm does not consider how to provide the robustness of the system under the condition of time dynamic change. In [39], the wireless communication problem of low-power Internet of Things for industrial equipment is analyzed, and an improved synchronous waveform monitoring algorithm is proposed, which can achieve better robustness under the environment of low-power Internet of Things. In [40], analysis the stochastic delay problem of wireless transmission in mine environment, and presents a clock synchronization method for wireless sensor networks based on underground tunnels in coal mines. A kind of evaluation node is added into the existing wireless sensor network. The distribution law of propagation delay can be obtained according to the behavior of sensing node. A clock synchronization method based on bidirectional message exchange mechanism is proposed in [41], which includes the clock synchronization mechanism of active node and overhearing node. This method has good performance under the assumption of Gaussian delay. Wang, Z. et al. [42] studies the problem of secure time synchronization in the industrial Internet of Things, effectively detects Sybil attacks in the network, and proposes a secure clock synchronization method based on timestamp correlation and node clock skew. In addition, there are many clock synchronization methods designed for delay, robustness and other performance, which is applied in different network topologies and industrial applications [43,44,45].

## 3. Model and Formulation

There are two kinds of nodes in the wireless sensor network: Base station node and Sensor node. The sensor node mainly collects and transmits the sensing data to base-station node, and then base-station node converges and transmits the data.

The clock synchronization scheme proposed in this paper is based on the underground mining construction environment, which includes many underground construction scenes, such as subways, tunnels, mines, etc. Among them, the situation of a mine is the most complex and the challenge is the greatest. The coal mine and metallic mine are the two most representative mine types, which have different tunnel structures. The structure of coal mine is composed of one or more long tunnels, which can be regarded as a chained structure. However, the situation of a metallic mine is much more complex, in which the structure is composed of irregular tunnels, and these tunnels are excavated according to the distribution of the veins. Thus, the overall structure of the metallic mine is a tree.

**Figure 1:**
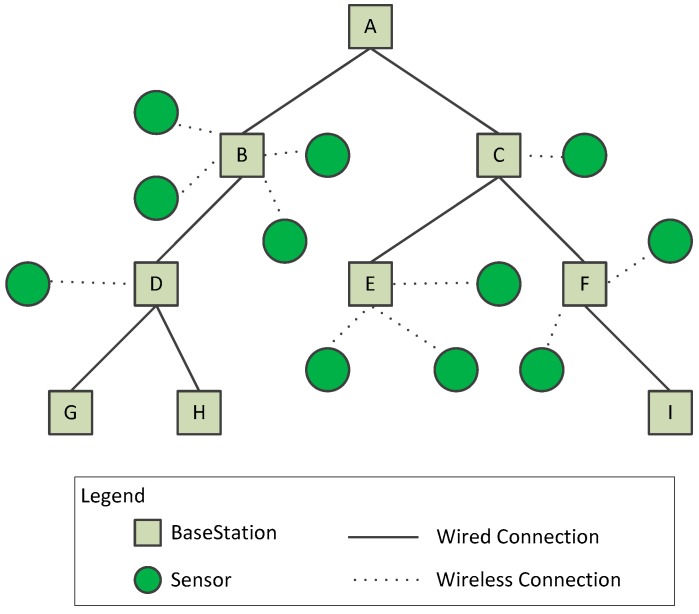
Topology of underground mining (metallic mine).

The mining of a metallic mine starts from the root node, for example, node  A in Figure 1. The mining work is carried out according to the distribution of veins. With the development of mining, the depth of roadways is deepening, and the number of branches is increasing, which forms a tree topology including multiple roadways. The deployment of wireless sensor network in this paper is based on this structure. Based on the length of the tunnel, one base-station deployed at certain intervals, and adjacent base-stations share a wired connection (and a wireless module is retained to ensure data transmission in the event of wired interruption). There are several sensors in one base-station, and these sensors collect different types of data, such as gases, temperatures, humidity, wind speeds, locator labels, and so on. The connection of the base-station and sensors is wireless.

In this paper, assume that there are a total of N base-stations to form a tree. For base-station  i (1≤i≤N), L(i) denotes the child nodes of base-station  i, and S(i) denotes all sensors of base-station i. Given N,L,S, we denote the above topology structure of underground mining as uTree(N,L,S).

Assuming that for any standard time t, C(t) denotes the local clock value of a sensor, this paper uses $\;C_{i,j}(t)$ to denote the local time values of *j*-th sensor in *i*-th base-station (denote $\;C_{i,0}(t)$ as base-station i). Compared with the standard time  t shown as following formulas:

(1) $$\;C_{i,j}(t)=\phi +\omega \times t+\varepsilon$$

We denotes ϕ as clock offset and ω as the clock skew of the reference node. ε is the synchronous noise, including transmission delay, data loss, and other related factors. In underground mining, in order to save energy, the crystal oscillator precision of all kinds of wireless equipment is low, such as the TI CC2530 module supporting the ZigBee protocol, whose maximum crystal oscillator frequency is 32 MHz, so it is difficult to calculate the value of clock skew between two different equipment accurately. Therefore, this paper sets the value of ω as 1. Similarly, ϕ and ε are difficult to estimate effectively in a certain synchronization process, especially in underground mining. Due to wireless transmission interference, packet loss rate, and other problems, usually, we cannot judge what causes the clock offset between two nodes, and we can only estimate the clock difference between two nodes. Therefore, in this paper, we also think of the value of ε as part of the clock offset. Thus, we mainly estimate the clock offset between clocks (close offset already includes the influence of ϕ and ε). That is:

∀i,j,p,q,t∈ℕ

Where

1≤i≤N,1≤j≤N,j∈L(i),p∈S(i),q∈S(j)

We have

(2) $$C_{i,p}(t)=\phi_{i,p}^{j,q}(t)+C_{j,q}(t)$$

We denote $\phi_{i,p}^{j,q}(t)$ as the clock offset of *p*-th sensor of *i*-th base-station and *q*-th sensor of *j*-th base-station. The goal of this paper is to erase the clock offset of all base-stations and sensors. That is:

(3) $$\phi_{i,p}^{j,q}(t)\;=\;C_{i,p}(t)-C_{j,q}(t)\;=\;0$$

However, in the actual engineering application environment, it is impossible to make the clock offset zero. Thus, this paper uses a tolerable upper bound ξ as a constraint of the clock offset. That is:

(4) $$|\phi_{i,p}^{j,q}(t)\;|\leq \xi$$

The value of ξ is determined according to different real-time requirements. Generally speaking, the real-time performance of the underground industrial internet of things is on the millisecond level, that is to say, the value of ξ is greater than 1 ms, considering the factor of energy consumption. In order to implement TDMA scheduling, we usually use timeslots to partition the superframe, and the size of each timeslot is determined according to the demand. In underground mining environments, the timeslot size is usually 3 to 10 ms. Therefore, the sum between ξ and the data transmission time can meet the requirements of TDMA scheduling as long as it is smaller than the size of one timeslot.

**Figure 2:**
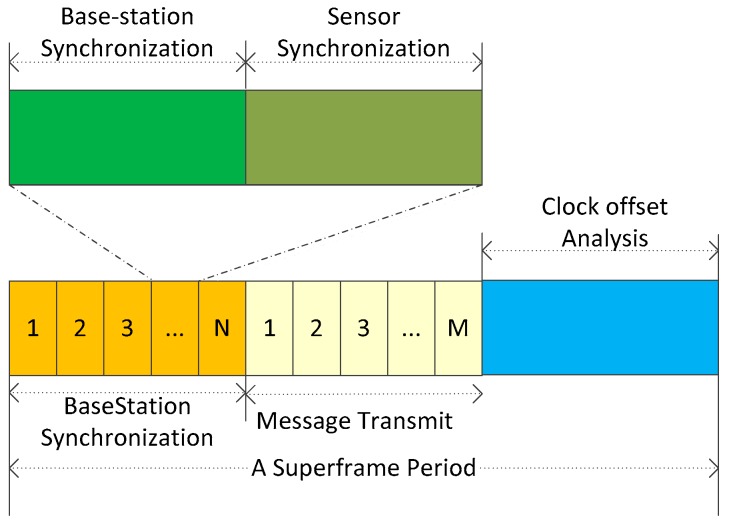
Superframe structure.

In this paper, a superframe with T timeslot is used to realize clock synchronization and TDMA data transmission. The superframe is divided into three parts: clock synchronization, data transmission, and clock offset analysis, as shown in Figure 2.

Assuming that N timeslots are required for clock synchronization and each timeslot contains two parts: base-station synchronization and sensor synchronization. There are M timeslots in the superframe for TDMA scheduling of wireless message transmission. The remaining T−N−M timeslot are used for clock offset analyzing. We use $t_{sync}$, $t_{analysis}$ to represent the time of N timeslot and the time of M timeslot. That is we complete synchronization at time $t_{sync}$ and start clock offset analyzing at time $t_{analysis}$. Thus, if the following condition is satisfied:

∀i,j,p,q∈ℕ

Where

1≤i≤N,1≤j≤N,j∈L(i),p∈S(i),q∈S(j)

In time $\;t_{sync}$ and $t_{analysis}\;$, we have

(5) $$|\phi_{i,p}^{j,q}(t_{sync})\;|\leq |\phi_{i,p}^{j,q}(t_{analysis})\;|\leq \xi$$

then we say the synchronization is successful.

ξ is the expected maximum clock error, that is, the clock error of any base-station and sensor cannot be greater than A from time $t_{sync}$ when synchronization is complete until time $t_{analysis}$.

**Figure 3:**
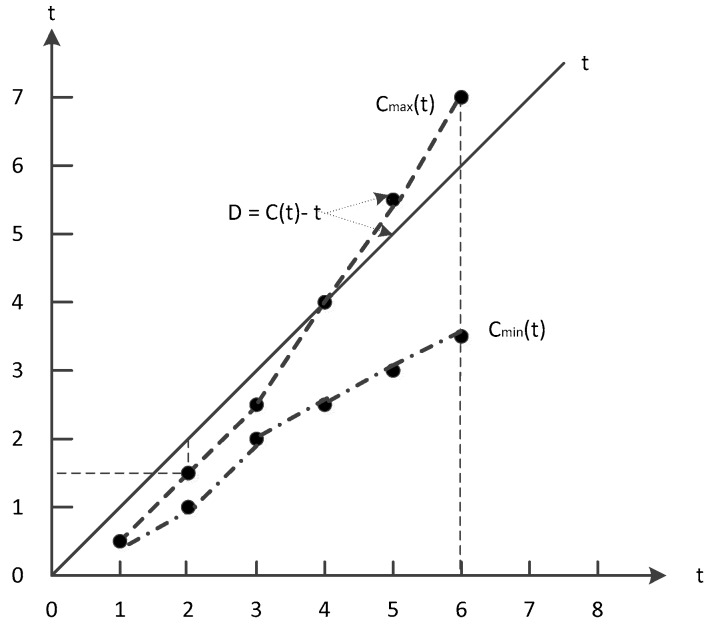
Clock decay.

Since the clock offset is influenced by hardware and environment, the clock offset varies randomly over time. So it is difficult to calculate accurately, but we can analysis the clock offset of one node within a time range of 1 to t. In this paper, we use $D_{i,p}(t)$ to represent the cumulative result of clock offset, which is called clock decay, shown in Figure 3.

∀i,p∈ℕ

Where 1≤i≤N,p∈S[i]

In time t, we have

(6) $$D_{i,p}(t)=C_{i,p}(t)-t$$

$\mathrm{MAX}(D_{i,p}(t)){|}_{i=1,t=t_{sync}}^{N,t_{analysis}}$ (1≤t≤tanalysis) is the maximum clock error, and there are generally two ways to handle this value:

(1) $\mathrm{MAX}(D_{i,p}(t)){|}_{i=1,t=t_{sync}}^{N,t_{analysis}}>\xi$

We will analyze the clock error values at *t –* 1, *t* + 1 of the node to determine whether it is a missing error due to a node failure. If it is a node failure (e.g., battery, hardware failure), the error of the node is ignored. Otherwise, an error correction strategy is employed to modify the error.

(2) $\mathrm{MAX}(D_{i,p}(t)){|}_{i=1,t=t_{sync}}^{N,t_{analysis}}\leq \xi$

The error of the node is calculated and the rate of change of the error is analyzed.

This paper completes synchronization at $t_{sync}$ and starts to analyze the clock offset at $t_{analysis}$. Thus, it is necessary to analyze the clock offset of all nodes from $t_{sync}$ to $t_{analysis}$. We denote $D_{analysis}$ as the clock decay rate, calculating the difference between the maximum and the minimum clock decay within $t_{sync}$ to $t_{analysis}$. That is:

∀i,p∈ℕ

Where 1≤i≤N,p∈S[i]

From time $t_{sync}$ to $t_{analysis}$, we have

(7) $$D_{analysis}=\frac{\mathrm{MAX}((\mathrm{MAX}(D_{i,p}(t))-\mathrm{MIN}(D_{i,p}(t))){|}_{i=1,t=t_{sync}}^{N,t_{analysis}})}{t_{analysis}-t_{sync}}$$

Apparently, Equation (7) is a pessimistic estimate, but using it to estimate the actual clock offset as a whole is undoubtedly valid. In order to ensure that clock offset synchronization meets Equation (5) constraints, it is necessary to adjust the period of the superframe according to the value of $D_{analysis}$ to guarantee the next superframe’s clock synchronization result.

We use θ to represent the time interval between $t_{sync}$ from $t_{analysis}$ of the next superframe. According to the value of $D_{analysis}$, if Equation (5) needs to be satisfied, the following condition must hold:

(8) $$\begin{array}{c} |\theta \times D_{analysis}|\leq \xi \\ \Rightarrow \theta \leq \frac{\xi }{\left|D_{analysis}\right|}\\ \Rightarrow \theta =\left\lceil \frac{\xi }{\left|D_{analysis}\right|}\right\rceil \end{array}$$

We denote $T^{\prime }$ as the adjusted period of the next superframe. We have:

(9) $$T^{\prime }=t_{sync}+\theta +T-t_{analysis}$$

## 4. Synchronization Scheme

Since the topology of this paper is a hybrid topology including tree-like and star-like networks, different synchronization algorithms should be designed for different topologies. According to the structure of the superframe, the clock synchronization process includes two parts: base-station to base-station and base-station to sensor. The base-station to base-station clock synchronization method belongs to sender-to-receiver (SRS), which starts from the root node of the tree topology and performs clock synchronization in pairs hierarchically. The base-station to sensor clock synchronization method includes sender-to-receiver (SRS) and receiver-to-receiver (RRS). The synchronization operation is initiated by base-station, and the goal is to ensure the clock synchronization between sender and sensor, as well as between base-station and sensor.

Clock synchronization begins with the first base-station, and when one base-station completes its synchronization of base-station to base-station, it begins to send a synchronization message to all the sensors to which it belongs to start the synchronization of base-station to sensor. When all base-stations and sensors are synchronized, the scheduling and transmission of wireless data begins, and the clock is reset in the next superframe.

### 4.1. Base-Station to Base-Station Synchronization

**Figure 4:**
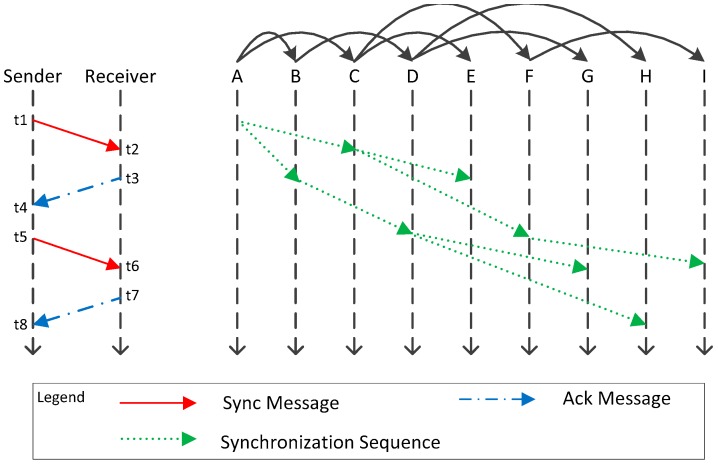
Base-station to base-station synchronization schematic.

Based on the tree topology of Figure 1, clock synchronization of base-station to base-station is initiated by the root node, and the hierarchical ergodic method is used to synchronize its child nodes in turn, as shown in the right part of Figure 4. Referring to TPSN algorithm, we use a sender-to-receiver model to synchronize, such as in the left part of Figure 4, One base-station as the sender node and its child base-station node as the receiver node. The sender node sends the synchronous message to the receiver at time *t*1, and the receiver node receives the message at time *t*2. Similarly, after three data transmissions, we get the time stamp *t*1, *t*2, *t*3, *t*4, *t*5, *t*6, *t*7, *t*8. We can estimate the approximation of clock offset of sender and receiver node based on the time stamp *t*1, *t*2, *t*3, *t*4.

Denote $\;\phi_{1}\;$ as the clock offset of sender to receiver, and $\;\phi_{2}\;$ as the clock offset of receiver to sender. 

We have:

(10) $$\phi_{1}=t2-t1$$

Similarly, we have:

(11) $$\phi_{2}=t4-t3$$

$\;\phi_{1}\;$ and $\;\phi_{2}\;$ are theoretically equal, but there is a difference in the actual program. In order to improve the synchronization accuracy, this paper adds a clock offset calculation, which is:

(12) $$\phi_{1}=t2-t1$$

For this reason, this paper uses the method of calculating the average to estimate the close offset between the two base-station nodes:

(13) ϕ=(ϕ1+ϕ2+ϕ3)3

According to Equation (1), the clock error between any two nodes includes clock skew, clock offset, and other factors, such as transmission delay. However, it is difficult to analyze each value in detail. Therefore, all the factors are analyzed synthetically in this paper, which leads to the result that the clock error analysis is not accurate at one time. The energy consumption will be increased and the delay will be larger if the analysis is carried out many times according to TPSN and other methods. Therefore, in order to minimize the transmission of synchronization messages and ensure the correctness of the synchronization results, this paper uses the average of the three results to evaluate the clock error.

**Figure 5:**
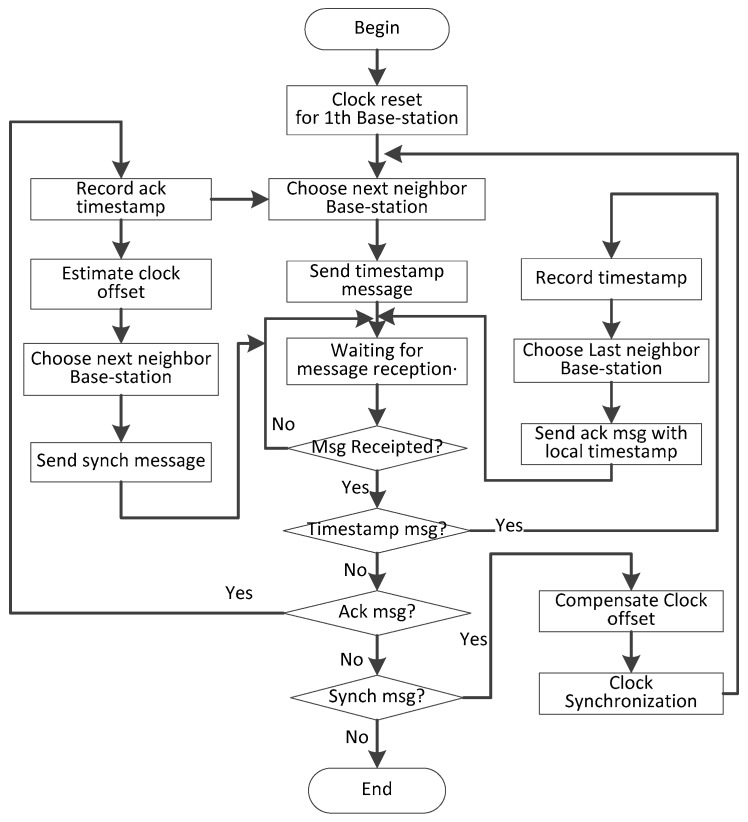
The flow chart of base-station synchronization.

The sender node sends message with ϕ as timestamp to the receiver at time *t5*. The receiver node receives the message at time *t*6, and then compensates the clock offset of its local clock according to Equation (2) using ϕ. At time *t*7, receiver node returns a synchronous acknowledgement message to sender node. If the sender node receives a synchronization acknowledgement message at *t*8, and the synchronization accuracy is verified according to the values of *t*7, ϕ, *t*8, and if the constraint of Equation (4) is satisfied, the synchronization is completed, otherwise a new synchronization process is executed again. The flowchart is shown in Figure 5.

According to the division of the superframe, after all base-stations have been synchronized, the scheduling of the data transmission is started, and the clock offset is analyzed in the last part of the superframe. Before the next superframe starts, the superframe is resized according to Equation (9).

If the communication is interrupted during the clock synchronization, such as insufficient battery power or equipment failure, the base-station that does not receive the synchronization message automatically adjusts the local clock according to the result of the clock offset analysis of the previous superframe, and after the adjustment is completed, the base-station begins to send the synchronization message to the subsequent node to ensure that the overall synchronization error is reduced as much as possible.

For any base-station, it first needs to complete the base-station to base-station synchronization and then perform synchronization with sensor, so the entire synchronization scheme is divided into two parts: function  uTree() and  uStar(). The function  uTree() executes on the base-station, completing the synchronization between base-stations, the pseudo code of which is shown in Algorithm 1.

**Algorithm1.** The clock synchronization algorithm for base-station to base-station. uTree(n,L(n)){ // *n* is one base-station, n∈[1,N] //L(n) is the children set of base-station *n*. // All base stations have been numbered according to a hierarchical traversal, starting with number 1. 1. start synchronization 2.  if (*n* = = 1) {// this base-station is the root of the tree, and it need to send the synchronization message first 3.   Send synchronization message with timestamp *t*1 to all base-station belonging to *L*(*n*); 4.  }// end if 5.  while (TRUE) {//start of while-loop wait to receive messages 6.   if (timestamp msg) { 7.    send ack message to sender node with timestamp $\phi_{1}$ and *t*3; 8.   }// end if 9.   else if (ackmsg) { 10.    send synch msg with timestamp $\phi_{2}$ and *t*5; 11.   }//end else if 12.   else {// synch msg 13.    Calculates the value of ϕ and adjusts the local clock; 14.    Send synchronization message with timestamp *t*1 to all base-station belonging to *L*(*n*); 15.     uStar(n,S(n)); // start the synchronization of base-station to sensor 16.    break while-loop; 17.   } // end else 18.  }// end while-loop 19.  end synchronization

### 4.2. Base-Station to Sensor Synchronization

**Figure 6:**
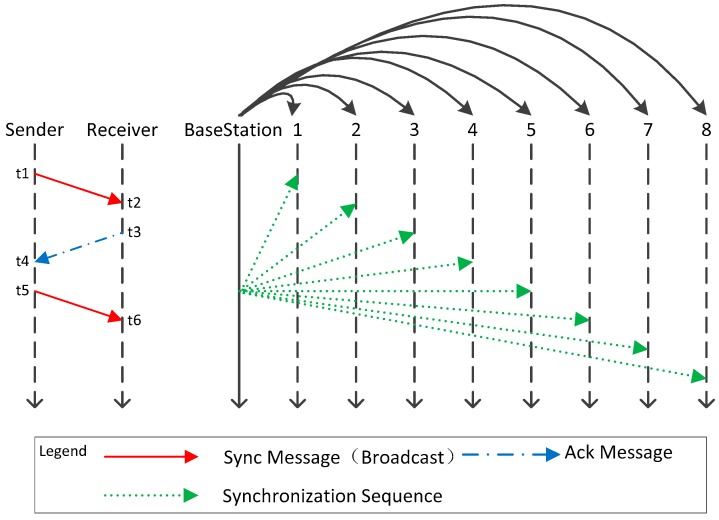
Synchronization schematic of the base-station to sensor.

The sensor in this paper is deployed near the base-station to form a star network centered on the base-station. When one base-station completes the clock synchronization of base-station to base-station, it is necessary to synchronize the sensors belonging to it to ensure that the data transmission between different sensors does not interfere. In this synchronization scheme, the base-station sends synchronization messages to all sensors belonging to it in broadcast mode, shown in the right part of Figure 6. When the sensors receive the synchronization message, it modifies the local clock according to the timestamp and returns a reply message.

Shown in the left part of Figure 6, the base-station serves as the sender and the sensor serves as the receiver. At time *t*1, the base-station broadcasts an echo message with *t*1 as the timestamp, and the sensor receives the message at time *t*2. It is assumed that all sensors receive echo messages from the base station at the same time (in the underground mining, this assumption is in line with the actual needs). All sensors return a reply message at time *t*3 and adjust its clock according to the value ϕ:

(14)  ϕ=t1+t3−t2+∂

∂ is a random number that represents the transmission delay between base-station and sensor, and according to Equation (4), ∂≤ξ.

At time *t*3, clock synchronization has been completed between all sensors, but the clock offset estimation between base-station and sensor is required. 

The base-station receives the message at time *t*4. Since different sensors have different values at *t*2, *t*3, we denote $CW_{i,j}$ as the received time of the message from sensor j to base-station i, and according to Equations (10) to (13),∀i,j,t1,t2,t3,t4∈ℕ,the clock offset of sensor j to base-station i is estimated as:

(15) ϕi,j=CWi,j(t2)−Ci,0(t1)+Ci,0(t4)−CWi,j(t3)2

This paper estimates the average clock offset of all sensors to base-station, that is:

(16) ϕ′=∑j=1count(s[i])ϕi,jcount(s[i])

**Figure 7:**
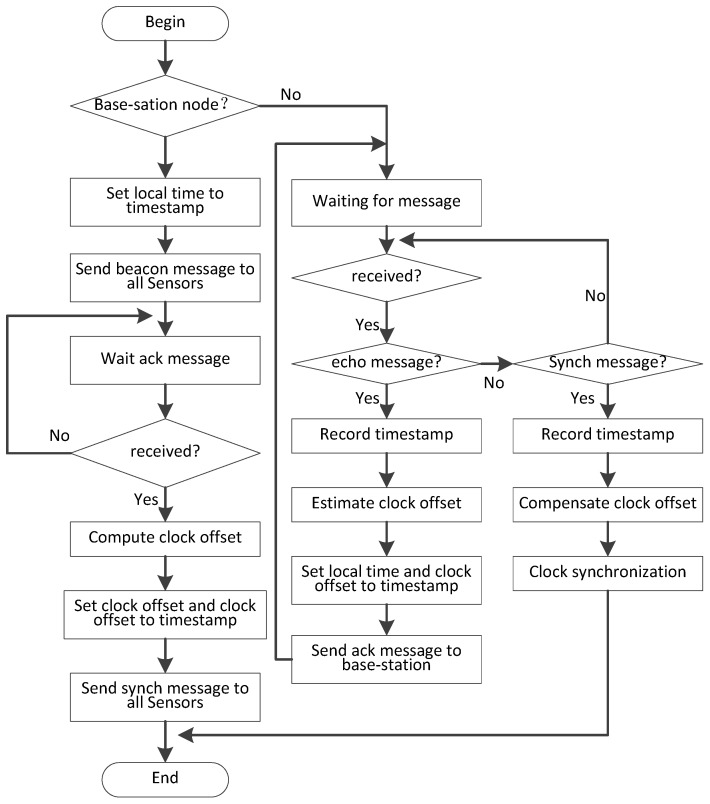
The flow chart of sensor synchronization.

At time *t*5, that base-station broadcast a synch message containing $\phi^{\prime }$ as a timestamp. The sensor receives the synch message at time *t*6 and adjusts the local clock according to the value of $\phi^{\prime }$.After time *t*6, the clock between base-station and all sensors has completed synchronization. The flowchart is shown in Figure 7.

For the base-station to sensor clock synchronization scheme, if an interrupt occurs for the communication message, it needs to distinguish the type of interrupt:

(1) If the base-station does not receive the ack message at *t*4, then the sender do not receive the echo message (or the sender has already received it, only the ack message is missing). The base-station determines whether synchronization needs to be restarted based on the number of missing nodes.

(2) If the synchronization deadline is exceeded and one sensor does not receive the echo message at time *t*2, the sender automatically adjusts based on the clock offset analysis of the previous superframe.

Since the sensor adjusts the local clock after the echo message is received, the clock error between the sensor and base-station is small even if the sync message is not received at time *t*6.

This paper uses the function  uStar() to perform synchronization between base-station and sensors, which are executed on the device of base-station and sensor, respectively. The clock synchronization scheme of base-station to sensor is initiated by base-station and the sensor listens for the sending of synchronization messages and performs synchronization operations.

The pseudo-code of the algorithm is shown in Algorithms 2 and 3.

**Algorithm 2.** The clock synchronization algorithm for base-station to sensor: Part A: base-station. uStar(n,S(n)){ // *n* is one base-station, n∈[1,N]
//S(n) is the list of all sensors belonging to base-station *n*. // All sensors have been numbered by base-station, starting with number 1. 1.  start synchronization 2.  Send echo message with timestamp *t*1 to all sensors; 3.  int r[*n*][] = 0; // r[*n*][*j*] is the clock offset of sensor *j* to base-station *n*; 4.  while (TRUE) {//start of while-loop, wait to receive messages 5.   if (ackmsg) {//received ack message 6.    Clock offset is calculated and logged in r[n][]; // according to Equation (15) 7.    if (count (S(n)) <= count(r[n][])){ //If all sender messages have been received 8.     Broadcast synch message to all sensors node with times tamp $\phi^{\prime }$; 9.     break while-loop; 10.    }// end if 11.   }// end if 12.  }// end while-loop 13.  end synchronization

**Algorithm 3.** The clock synchronization algorithm for base-station to sensor: Part B: sensor.uStar(n,j){ // *n* is the base-station, n∈[1,N] //is the sensor number. 1.  start synchronization 2.  while (TRUE) {//start of while-loop, wait to receive messages 3.   if (echo msg) {//received echo message from base-station 4.    Send ack message to base-station *n* with stamp *t*2, *t*3; 5.    Adjust local clock as ϕ; //according to Equation (14) 6.   }// end if 7.   else {//received synch message from base-station 8.    Adjust local clock as $\phi^{\prime }$ ;//according to Equation (16) 9.    break while-loop; 10.   }// end if 11.  }// end while-loop 12.  end synchronization

## 5. Evaluation

In this section, we conduct an experiment for the synchronization scheme of WSNs in underground mining proposed in this paper.

In the environment of Industrial Internet of Things, especially in the underground construction environment, the wireless data transmission and reception in wireless clock synchronization faces great challenges due to electromagnetic radiation, irregular tunnel structure, and limited energy consumption. Therefore, it is difficult to accurately evaluate the performance of the algorithm in simulation experiments [46,47,48]. Therefore, in order to improve the accuracy of the experimental results as much as possible, embedded hardware equipment is used to carry out the experiment, and according to the underground topology, build the base-station and sensor experimental environment. At present, the main short-range wireless communication protocols are mostly based on IEEE 802.15.4, such as Wireless Hart, ZigBee, and so on. Therefore, this paper chooses the CC2530 communication module of TI Company (Texas Instruments, 12500 TI Boulevard Dallas, Texas 75243 USA) to carry on the experiment. The CC2530 is a true system-on-chip (SoC) solution for IEEE 802.15.4, Zigbee, and RF4CE applications. It enables robust network nodes to be built with very low total bill-of-material costs. The CC2530 combines the excellent performance of a leading RF transceiver with an industry-standard enhanced 8051 MCU, in-system programmable flash memory, 8-KB RAM, and many other powerful features. The CC2530 comes in four different flash versions: CC2530F32/64/128/256, with 32/64/128/256 KB of flash memory, respectively. The CC2530 has various operating modes, making it highly suited for systems where ultralow power consumption is required. Short transition times between operating modes further ensure low energy consumption.

**Figure 8:**
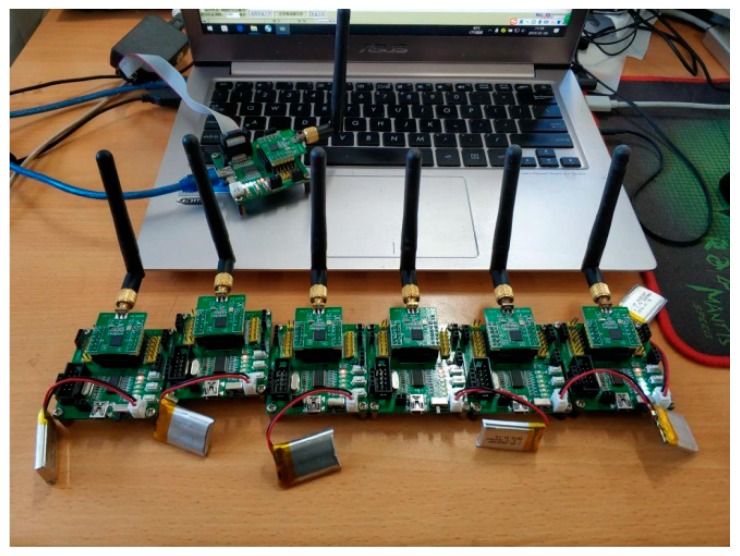
The diagram of the experimental environment.

As shown in Figure 8, this paper uses the CC2530 communication module to carry out the experiment. Among them one CC2530 serves as the base-station, connects to the notebook computer through USB, sends the related data to the notebook computer through the serial port, and carries on the collection of the experiment data, at the same time, six CC2530 modules serve as sensors. In this paper, there are 35 CC2530 s, which consist of five base-stations, with each base-station containing six sensors. 

The experiment settings information is shown in Table 1. The peak rate of the CC2530 is 250 kbps, but this is rarely achieved in practice. In this paper, We carry out our experiment in the laboratory, and the wireless transmission rate was20–30 kbps. At the same time, by setting lower power, it was nearly possible to ensure that the distance between the different base-station in accordance with the actual environment under the mine. The CC2530 has an effective power range of −28 dBm to +4.5 dBm, with a default of 1 dBm, and has been tested for transmission distances of up to 400 meters in an open environment. In order to build the test environment in the laboratory, it is necessary to reduce the transmission distance of the CC2530. Therefore, this paper sets the power to −8 dBm; the corresponding output current is 25 mA, after testing, the transmission distance is about 20 m. We use a hierarchical tree structure to simulate the underground environment. We deploy a base-station in two rooms, which are close to each other, and have many Wi-Fi equipment in the room, and some communication equipment which transmission bands below 1 GHz, such as a CC1101, etc. To reflect the impact of energy consumption on clock synchronization, all CC2530 used herein are battery powered.

Due to the special hybrid network topology, two different clock synchronization methods are needed, and the existing methods are difficult to be directly applied. The main reasons include: 

(1) Most of the existing methods do not consider the special network topology, and generally consider the clock synchronization between two nodes (or simple topology of one-to-many nodes). The clock synchronization in this paper needs to start from the root node to traverse the tree topology to achieve clock synchronization in turn.

(2) Due to the particularity of the underground Industrial Internet of Things, the clock synchronization in this paper needs to consider the energy consumption and the performance of the processor.

In this paper, the synchronization accuracy and energy consumption are analyzed. At the same time, a comparative analysis of robustness is made in this paper. We test the performance of the scheme presented in this paper, and the main comparative algorithm are: TPSN [16], LTS [25], and RBS [15].

TPSN is a classic method of wireless clock synchronization between two nodes. This method has high synchronization accuracy in ideal experimental environment, but it does not consider the problem of energy consumption. The method in this paper is based on the improvement of TPSN method and is applied in the tree topology. Thus, we choose this method to reflect the performance of the algorithm in synchronization accuracy. LTS is an improved TPSN method, which considers the energy consumption problem and sacrifices part of the synchronization accuracy. This paper chooses this method to compare the energy consumption performance of the method in this paper. Similarly, RBS is a typical receiver clock synchronization algorithm; this paper chooses this method to compare the performance of the method of base-station to sensor.

In the experiment of synchronization accuracy, we test the synchronization scheme of base-station to base-station and base-station to sensor, which named B-Sync and S-Sync, respectively.

### 5.1. Synchronization Accuracy Test

**Figure 9:**
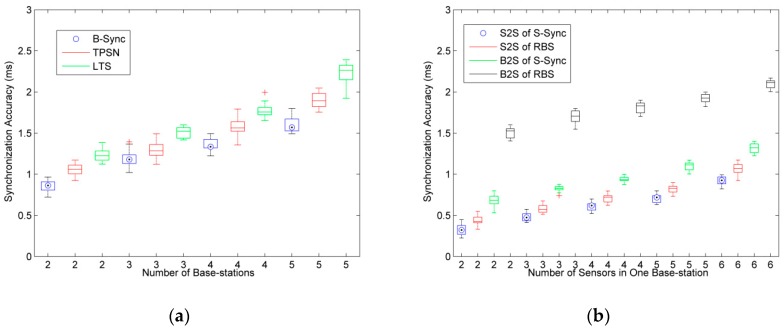
Comparative analysis of synchronization accuracy for base-station to base-station synchronization. (**a**)Base-station to base-station synchronization; and (**b**) base-station to sensor synchronization.

In the synchronization accuracy test of B-Sync, we compare the classical algorithms TPSN and LTS to observe the change of the clock synchronization accuracy in the case of different base-station nodes, as shown in Figure 9a.

In order to improve the accuracy of the experimental results, the experiment was carried out 20 times each, and the data are presented in box plots. According to the results of the experiment in Figure 9a, as the number of base-station nodes increases, clock synchronization accuracy continues to increase, that is, the clock offset of any two base-station increases. This is because the global clock synchronization scheme is adopted in this paper: when all base-stations are synchronized, we compute the maximum value of clock offset between any two base-stations. So with the increasing number of base-stations, the time required for clock synchronization is increased, which leads to a clock offset for the base-station that has been synchronized in the subsequent base-station synchronization. The longer the synchronization time, the larger the value of close offset.

Compared with the TPSN and LTS methods, the B-Sync method designed in this paper can achieve lower clock synchronization accuracy. In this paper, we have tested up to five base-stations. In this case, the maximum clock synchronization accuracy of B-Sync can be controlled within 1.8 ms, far less than 2.0 ms of TPSN and 2.3 ms of LTS. Moreover, in practical engineering applications, a timeslot is usually set to 3 ms to 10 ms. Therefore, the B-Sync method designed in this paper can meet the synchronization requirements of wireless sensor networks in underground mining.

More importantly, in the tree topology of underground mining, we are always concerned about the synchronization accuracy between nearer base-stations, which is because having no interference from wireless communication for base-stations farther away and the farther base-station without communication interference is not considered in the design of the TDMA algorithm. Therefore, the B-Sync method proposed in this paper can provide lower synchronization accuracy in practical engineering applications.

In the synchronization accuracy experiment of base-station to sensor, the RBS method is used to compare with S-Sync. In this paper, we test two kinds of clock synchronization accuracy: base-station and sensor (B2S), and sensor and sensor (S2S).

According to the experimental results of Figure 9b, with the increase of the number of sensors, the accuracy of clock synchronization tends to increase. However, because both S-Sync and RBS methods adopt the method of base-station broadcasting to realize the synchronization for all sensors, the number of sensors nodes has less effect on the synchronization result.

Compared with the RBS method, the S-Sync method in this paper can guarantee the lower clock synchronization accuracy, especially the B2S synchronization accuracy. Since the RBS method is lacking the synchronization method of base-station and sensor, the clock error caused by the data transmission delay is large.

In practical engineering applications, the TDMA scheduling of the sensor can be done by base-station. Therefore, the accuracy of clock synchronization between the sensor and base-station has little effect.

### 5.2. Energy Consumption Test

In the wireless sensor network, the node consumes the energy module mainly includes the sensor module, the data processor module, the wireless communication module. From the perspective of wireless sensor network operation, the operation of energy consumption is mainly divided into two types: communication-related energy consumption and non-communication-related energy consumption. The energy consumption related to communication mainly focuses on the energy consumption of the sensor module to collect data and the data processing module to process data. Usually, the sensor node collects less information and the data processing is simple, so the sensor module and the data processing module have lower power consumption. Most of the energy consumption of the node is in the wireless communication module to realize the operation of data transmission between nodes.

Therefore, this paper uses the number of synchronous message to represent the energy consumption, and tests the energy consumption of B-Sync and S-Sync schemes under different conditions.

Different clock synchronization accuracy has different requirements for energy consumption. Therefore, the clock synchronization accuracy upper bond ξ of Equation (5) is tested separately to test the average energy consumption of nodes in different number of base-stations and sensors.

**Figure 10:**
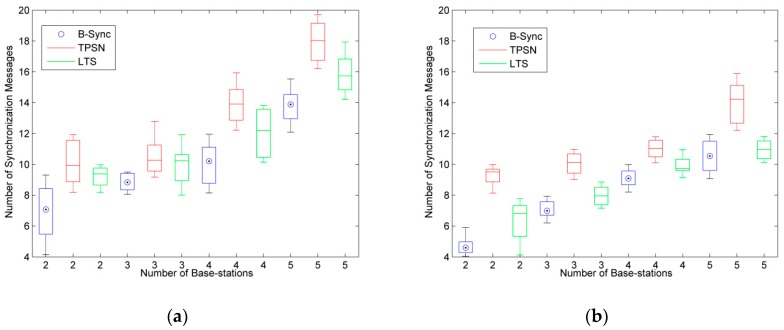
Comparative analysis of energy consumption for base-station to base-station synchronization. (**a**) ξ ≤ 1 (ms); (**b**) 1 < ξ ≤ 2 (ms).

As shown in Figure 10, in the energy consumption test of the B-Sync scheme, we analyze the number of synchronization messages for B-Sync, TPSN, and LTS for different synchronization accuracy (ξ≤1 and 1 < ξ ≤ 2) under the condition of two to five base-stations, respectively. In order to achieve the corresponding synchronization accuracy, it needs to compensate for the clock offset by sending multiple synchronization messages. According to the experimental results of Figure 10, with the increase of synchronization accuracy, the number of synchronization messages is decreased, while the number of synchronization messages is less in B-Sync method under the same conditions, which shows that the energy consumption of the method designed in this paper is lower. In the case of five base-stations, B-Sync reduced energy consumption by approximately 11% and 14% compared to LTS and TPSN, respectively, with the same accuracy.

**Figure 11:**
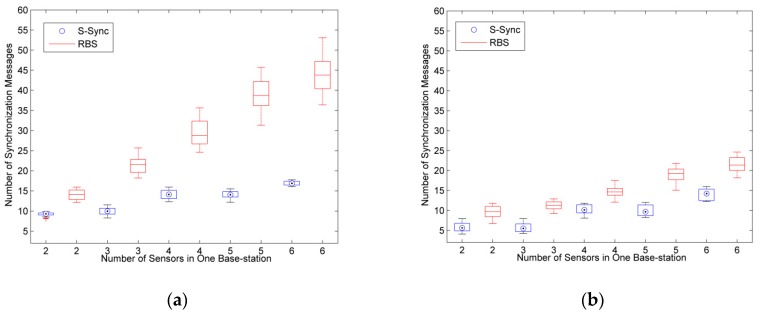
Comparative analysis of energy consumption for bases-station to sensor synchronization. (**a**) ξ ≤ 0.5 (ms); (**b**) 0.5 < ξ ≤ 1 (ms).

In the energy consumption test of S-Sync scheme, we test the number of synchronous messages for S-Sync and RBS for different number of sensors, in different clock synchronization accuracy (ξ ≤ 0.5 and 0.5 < ξ ≤ 1) under the condition of two to six sensors in one base-station respectively. The result is shown in Figure 11. According to the experiment results, the number of synchronous messages in S-Sync and RBS increases as the number of sensors increases, but the number of synchronous messages in S-Sync is significantly less than that in RBS, and this gap becomes more pronounced when the number of sensors is large. This is because S-Sync sends synchronization timestamp messages to the base-station, while RBS requires sensors to send synchronous messages to each other. Obviously, when the number of nodes is large, the energy consumption of RBS algorithm is very high. Compared with the S-Sync method, the energy consumption of RBS is increased by at least 20%, and the maximum energy consumption is 60%.

### 5.3. Robustness Test

**Figure 12:**
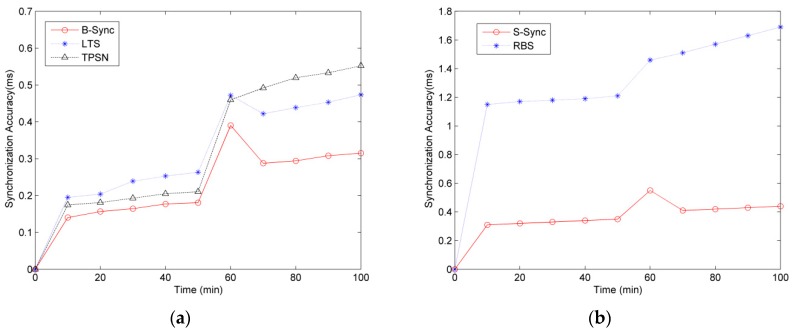
Comparative analysis of robustness. (**a**)Base-station to base-station synchronization; and (**b**) base-station to sensor synchronization.

During the robust experiment of B-Sync, we observe the synchronization accuracy of B-Sync, TPSN and LTS algorithms changing with time, as shown in Figure 12a.We select the maximum absolute value of any two clock offsets in all base-stations as the overall clock synchronization accuracy, and set the period of the superframe of 50 min. Thus, the system will resynchronize the clock at the 50th minute, and we randomly select one base-station, interrupt its power supply, and observe the change of the synchronization accuracy of the two methods in the following time. We calculate the average results of 10 experiments. As shown in Figure 12a, the robustness of B-Sync is better than that of TPSN and LTS. Especially when the communication is interrupted, the TPSN and LTS methods cannot synchronize the clock effectively, and the synchronization accuracy is increased.

In S-Sync’s robustness experiment, we mainly observe the change of the synchronization accuracy between base-station and sensor because the synchronization accuracy between sensors is determined by the synchronization message of the base-station, and the hardware performance between sensors is basically the same, which leads to the stable linear growth of the synchronization accuracy change between them with the change of time, and the influence of communication interruption and other factors is less.

We chose seven CC2530s for the experiment, including one base-station and six sensors. Similarly, we set the superframe period to 50 minutes and interrupt communication between the base-station and sensors at the 50th minute to observe changes to the synchronization accuracy, as shown in Figure 12b.

According to the experimental results of Figure 12b, the robustness of S-Sync is better than that of the RBS algorithm. Since the RBS algorithm does not guarantee the synchronization accuracy between the base-station and sensor, the synchronization error of the RBS algorithm is larger than that of S-Sync. When the communication is interrupted, the RBS algorithm cannot send the synchronization message to the sensors. Therefore, the synchronization error will be very large, and the error will increase gradually with the increase of time. The S-Sync method includes a mechanism for handling communication interrupts so that when communication interrupts occur all sensors automatically adjust local time, although synchronization errors still occur but, compared to RBS, this synchronization error is already within the range that can be received, especially in the wireless sensor network of the underground mining construction environment.

## 6. Conclusions

This paper presents the clock synchronization scheme of wireless sensor networks in an underground mining environment. The nodes of wireless sensor networks are divided into base-station and sensor. According to the characteristics of underground mining tunnel structures, the base-station is formed into a tree structure, and each base-station and its sensors form a star network. According to the topology, we design two synchronization schemes: base-station to base-station and base-station to sensor, using the strategy of dynamic super-frame size to ensure that the system can adjust the synchronization time according to the situation. Finally, the synchronization mechanism proposed in this paper is tested and analyzed. The analysis results show that, under the same conditions, this method can ensure less clock synchronization error, and can effectively reduce energy consumption. At the same time, in the special case, such as a communication interruption, the synchronization method proposed in this paper can guarantee better robustness.

## Figures and Tables

**Table 1:** Experiment settings.

Experiment hardware:	TI CC2530 F256
MAC protocol:	TDMA
Number of RF channels:	Single channel
Communication rate:	20–30 kbps
The output power of the transmitter:	−8 dBm
Number of base-stations:	2, 3, 4, 5
Number of sensors per base-station:	2, 3, 4, 5, 6
Duration of a timeslot:	2 ms
Default superframe size:	300,000 timeslots
Duration of each experiment trial	6 superframes
Experiment trials for each configuration:	20
